# Does modeling causal relationships improve the accuracy of predicting lactation milk yields?

**DOI:** 10.3168/jdsc.2022-0343

**Published:** 2023-07-21

**Authors:** Xiao-Lin Wu, Asha M. Miles, Curtis P. Van Tassell, George R. Wiggans, H. Duane Norman, Ransom L. Baldwin, Javier Burchard, João Dürr

**Affiliations:** a Council on Dairy Cattle Breeding, Bowie, MD 20716; b Department of Animal and Dairy Sciences, University of Wisconsin–Madison, Madison, WI 53706; c USDA, Agricultural Research Service, Animal Genomics and Improvement Laboratory, Beltsville, MD 20705

## Abstract

This study compared 3 correlational (best prediction, linear regression, and feed-forward neural networks) and 2 causal models (recursive structural equation model and recurrent neural networks) for estimating lactation milk yields. The correlational models assumed associations between test-day milk yields (health conditions), while the casual models postulated unidirectional recursive effects between these test-day variables. Wood lactation curves were used to simulate the data and served as a benchmark model. Individual Wood lactation curves provided an excellent parametric interpretation of lactation dynamics, with their prediction accuracies depending on the coverage of the lactation curve dynamics. Best prediction outperformed other models in the absence of mastitis but was suboptimal when mastitis was present and unaccounted for. Recurrent neural networks yielded the highest accuracy when mastitis was present. Although causal models facilitated the inference about the causality underlying lactation, precisely capturing the causal relationships was challenging because the underlying biology was complex. Misspecification of recursive effects in the recursive structural equation model resulted in a loss of accuracy. Hence, modeling causal relationships does not necessarily guarantee improved accuracies. In practice, a parsimonious model is preferred, balancing model complexity and accuracy. In addition to the choice of statistical models, the proper accounting for factors and covariates affecting milk yields is equally crucial.

## Highlights

• This study compared correlational and casual models for estimating lactation milk yields.
• Disparities in the goals and scenarios for correlational versus casual models are elucidated.
• The accuracy was demonstrated through the decomposition of expected prediction error.
• The recurrent neural networks yielded the highest accuracy when mastitis was present.
• A parsimonious model balanced model complexity and accuracy.

## Graphical Abstract

**Figure:**
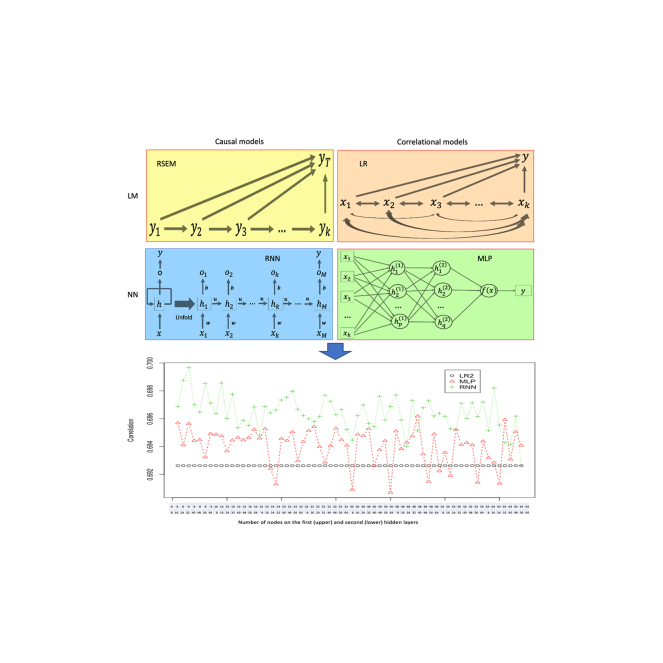
**Summary:** An early milk yield or health condition can affect subsequent yields or conditions. Hence, recursive models can be useful for describing lactation dynamics. This study compared 3 correlational and 2 causal models for estimating lactation milk yields. Best prediction outperformed other models in the absence of mastitis, but was suboptimal when mastitis was present and unaccounted for. Recurrent neural networks achieved the best accuracies when mastitis was present. Causal models facilitated the inference about the causality underlying lactation, but precisely capturing the causal relationships was challenging due to the complexity of the underlying biology. Misspecification of recursive effects in the recursive structural equation model led to a loss of accuracy. Therefore, modeling causal relationships does not necessarily guarantee improved accuracies. In practice, a parsimonious model is preferred, balancing between model complexity and accuracy. In addition to choosing appropriate statistical models, it is equally crucial to account for factors and covariates affecting milk yields.

Various mathematical methods have been proposed to estimate lactation milk yields, including empirical methods (McKellip and Seath, 1941; McDaniel, 1969), best prediction (**BP**) (VanRaden, 1997; Cole et al., 2009), and feed-forward neural networks (**FNN**) (e.g., Salehi et al., 1998; Grzesiak et al., 2003; Njubi et al., 2010). Most of these methods, except for the empirical ones, primarily assume associations (or correlations) between daily or test-day milk yields (**DMY**; see graphical abstract). However, statistically, an association does not necessarily indicate any underlying causality relationship. In reality, an early milk yield or health condition can affect a subsequent yield or condition, indicating that recursive models may be beneficial for predicting lactation dynamics (van Bebber et al., 1999). This study compared the performance of 3 association models (i.e., BP, linear regression [**LR**], and FNN) and 2 causal models (i.e., recursive structural equation model [**RSEM**] and recursive neural networks [**RNN**]) for estimating lactation milk yields.

The Wood lactation curve (**WLC**; Wood, 1967) was used to simulate actual daily milk yields for 3,000 cows (e.g., Figure 1A) and served as a benchmark model:

[1] $$y_{t}=at^{b}e^{-ct},$$

where *y_t_* was the yield on time *t* in days. The model parameters were sampled from *a* ~ *TN* (9.77, 2.23^2^), *b* ~ *TN* (0.18, 0.06^2^), and *c* ~ *TN* (0.004, 0.0007^2^), where *TN* stands for a truncated normal distribution with all nonnegative values. Residuals (*e_i_*) were simulated from $e_{i}∼N\left(0,\frac{1-\rho }{\rho }\sigma_{p}^{2}\right),$ mimicking within-test-day repeatability (*ρ*) as if a daily milk yield could be repeatedly measured on a given day, where $\sigma_{p}^{2}$ was the actual phenotypic variance, and *ρ* = 0.5, 0.7, and 0.9, respectively. The observed yield for each animal equaled the actual yield plus a residual. In the presence of mastitis, SCS were linearly proportional to the actual daily milk yields on the same test date and rescaled within 0 and 9. When SCS exceeded the subclinical threshold (say, 5), it negatively affected the following milk yield with a decreasing factor equal to 1.0 − 1.5*^scs^*/39. Mathematically, the incomplete Gamma function of WLC imposed dependencies between daily milk yields, determined by the 3 model parameters. The closer the milking days, the larger the (partial) correlations (Figure 1B). It is important to note that this study simulated typical or standard lactation curves. However, reality can be more complex, involving atypical (i.e., continuously decreasing) and other nonstandard types of curves (Macciotta et al., 2005).

**Figure 1:**
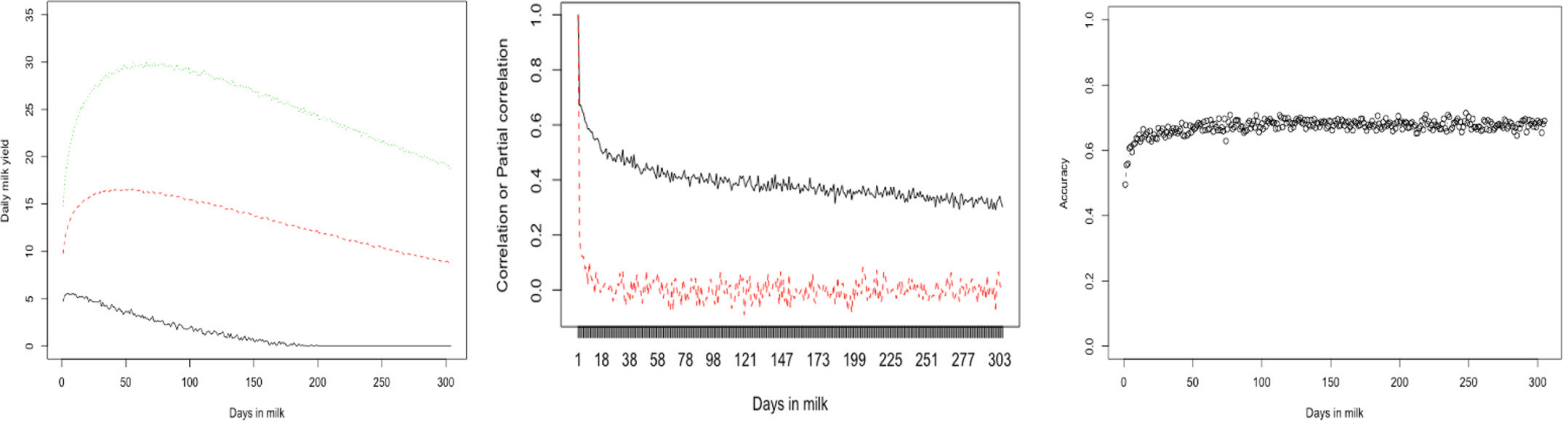
Illustration of mean and 95% interval of simulated daily milk yields with a 0.70 repeatability (left), correlations (solid black line) and partial correlations (dotted red line) between milking day 1 and the following milking days as the milking interval increased (middle), and predictive R^2^ accuracies of estimated daily milk yields based on linear regression without mastitis (right).

The BP approach is the de facto method currently used for estimating lactation milk yields in the United States (VanRaden, 1997; Cole et al., 2009). Let *y* be a random variable for an unobserved daily milk yield, expressed as a deviation from the corresponding population mean and predicted by some function *f*(**z**) of a random variable vector **z** for the observed test-day milk yield deviations. Define the “best predictor” as that for which the expected value $E\left\{{\left[y-f\left(z\right)\right]}^{2}\right\}$ is a minimum. Then, the “best choice” for *f*(**z**) is the following:

[2] $$\begin{array}{c} f\left(z\right)=E(y|z)\\ =cV^{-1}z, \end{array}$$

where **c** is a vector of the covariances between *y* and **z**, and **V** is a variance-covariance matrix of **z**; both are assumed to be known. Adding the corresponding population mean to a deviation gives an estimated daily milk yield. The total (305-d) lactation yield can be estimated by aggregating all the observed and estimated daily milk yields for each cow. Alternatively, the 305-d milk yield can be calculated directly using [2] without estimating unobserved daily milk yields. The implementation of BP followed VanRaden (1997), except the means of daily (or lactation) milk yields were taken from the simulated values.

Including additional fixed effects in [2] resulted in a linear model. That is,

[3] $$y=1\mu +X_{1}\beta_{1}+X_{2}\beta_{2}+e,$$

where *µ* is the overall mean, **y** is a vector of test-day yield, **β**_1_ is a vector of fixed effects of test day yield effects, **β**_2_ is a vector of fixed effects of SCS, **X**_1_ and **X**_2_ are the corresponding incidence matrixes, and **e** is a vector of residuals.

An RSEM is also a linear regression model, except that it additionally postulates unidirectional, recursive effects (Gianola and Sorensen, 2004) from a preceding milking variable (*k*) to the following milking variable (*j*), denoted by λ*_jk_*. The structural coefficient matrix defining the recursive relationships is the following:

[4] $$\Lambda =\left(\begin{array}{ccccccc} 1 & 0 & 0 & \cdots & 0 & 0 & 0\\ -\lambda_{21} & 1 & 0 & \cdots & 0 & 0 & 0\\ 0 & -\lambda_{32} & 1 & \cdots & 0 & 0 & 0\\ \vdots & \vdots & \vdots & \ddots & \vdots & \vdots & \vdots \\ 0 & 0 & 0 & \cdots & -\lambda_{k\left(k-1\right)} & 1 & 0\\ -\lambda_{T1} & -\lambda_{T2} & -\lambda_{T3} & \cdots & -\lambda_{k\left(k-1\right)} & -\lambda_{Tk} & 1 \end{array}\right),$$

where *T* is the total number of dependent variables in the model. The RSEM was implemented via the Markov chain Monte Carlo simulation (Wu et al., 2007).

A feed-forward, multilayer perceptron (**MLP**) consists of at least 3 layers of nodes: an input layer, a hidden layer, and an output layer. For example, an MLP with a single hidden layer for regression is mathematically defined as follows:

[5] $$y=\varphi \left(\sum_{k=1}^{K}w_{k}^{\left(2\right)}h\left(\sum_{j=1}^{T}w_{kj}^{\left(1\right)}x_{j}+w_{k0}^{\left(1\right)}\right)+w_{0}^{\left(2\right)}\right).$$

In the above, *x*_1_,…, *x_T_* are inputs (e.g., observed DMY), $w_{k0}^{\left(1\right)}$ and $w_{kj}^{\left(1\right)}$ are the bias and weights for the input layer, respectively, $w_{0}^{\left(2\right)}$ and $w_{k}^{\left(2\right)}$ are the bias and weights, respectively, for the hidden layer, and φ and *h* are activation functions. Here, *k* = 1, …, *K* indexes each hidden function transformation unit. We omitted the index for individual animals for the simplicity of mathematical notations. Briefly, the MLP works as follows. First, an MLP constructs *k* linear combinations of the inputs, $\;a_{k}=\sum_{j=1}^{T}w_{kj}^{\left(1\right)}x_{j}+w_{k0}^{\left(1\right)}.$ Then, each is transformed using a differentiable, nonlinear activation function in the hidden layer, also known as activations, that is, *h_k_* = *h*(*a_k_*). Finally, the output unit activations are transformed by the activation function to give the network output $y=\varphi \left(\sum_{k=1}^{K}w_{k}^{\left(2\right)}h_{k}+w_{0}^{\left(2\right)}\right).$ The above MLP expands naturally to have multiple hidden layers, usually interconnected in a feed-forward way.

Recursive neural networks are deep neural networks created in learning sequence data structures, hence appealing to model dynamic lactation data. In an RNN, the input consists of data in sequence: $X=c\left(\begin{array}{ccc} \begin{array}{cc} x_{1} & x_{2} \end{array} & \begin{array}{cc} \ldots & x_{t} \end{array} & \begin{array}{cc} \ldots & x_{T} \end{array} \end{array}\right)$ for *t* = 1, …, *T*, where ${\text{x}}_{\text{t}}^{'}=\left(x_{t1}\ldots x_{tp}\right)$ is a vector of input data (i.e., test-day yield and SCS when applicable) at the *t*th test date. The RNN processes the input sequence, one vector at a time, say **x***_j_*. Then, the network updates the activations *h_t_* in the hidden layer, taking as input the vector **x***_t_* and the activations *h_t_*_−1_ from the previous step in the sequence. Again, consider a single hidden layer with *K* units. For the *t*th test date, the activations, say, at the *k*th hidden layer unit, are updated as follows:

[6] $$h_{tk}=h\left(w_{k0}+\sum_{j=1}^{p}w_{kj}x_{tj}+\sum_{k^{\prime }=1}^{K}u_{kk^{\prime }}h_{t-1,k^{\prime }}\right),$$

where $w_{k0}$ and $w_{kj}$ are the bias and weights defined for the input data at the *t*th test date processed by the *k*th unit in the hidden layer, and $u_{kk^{\prime }}$ is the weight defined for the hidden-to-hidden layers. The corresponding output for the *t*th test date is then computed as

[7] $$o_{t}=b_{0}+\sum_{k=1}^{K}b_{k}h_{tk},$$

where *b*_0_ and *b_k_* are the bias and weights defined for the output layer. This way, the output at the *t*th test date combines information from the prior test dates up to time *t* − 1 and the current inputs from the current test day at time *t* (see graphical abstract). The model parameters are obtained by minimizing the following loss function for an observation (**X**, *y*), where *i* = 1, …, *n* for animals.

[8] $$\begin{array}{l} L=\sum_{i=1}^{n}{\left(y_{i}-o_{iM}\right)}^{2}\\ =\sum_{i=1}^{n}{\left(y_{i}-\left(b_{0}+\sum_{k=1}^{K}b_{k}h\left(w_{k0}+\sum_{j=1}^{p}w_{kj}x_{iTj}+\sum_{k^{\prime }=1}^{K}u_{kk^{\prime }}h_{i,T-1,k^{\prime }}\right)\right)\right)}^{2}. \end{array}$$

Training a long-sequence RNN can be challenging because of the vanishing or exploding gradient problems. Hence, more sophisticated activation functions were used instead, such as long short-term memory (**LSTM**) units (Hochreiter and Schmidhuber, 1997).

In this study, the FNN consisted of 1 input layer, 2 hidden layers, and 1 output layer. Backpropagation was used for training, and the weights were obtained by minimizing the error function (Rumelhart et al., 1986). L2 regularization (l = 0.1) was applied to the kernel weights matrix. A modified RNN was implemented by the “keras” R package, which consisted of an LSTM unit layer for processing the sequential DMY, 2 hidden layers, and an output layer. For FNN and RNN, rectified linear (ReLu) activation functions were used in hidden layers, and no activation function was defined for the output layer. Early stopping was imposed up to 100 rounds or when the loss function (mean squared errors) did not improve. Based on preliminary runs, we chose 64 and 32 neurons for the 2 hidden nodes. Model performance was evaluated using holdout cross-validations (**HCV**) with 10 replicates. For BP, LR, and RSEM, 2,400 animals (80%) were used for training and 600 animals (20%) for testing in each replicate. For the 2 NN models, 1,950 (65%) animals were used for training, 450 animals (15%) were used for validation, and 600 animals (20%) were used for testing. Predictive R^2^ accuracy was calculated in the testing sets.

Wood lactation curve was used to simulate the data because of its excellent parametric interpretation of the lactation dynamics. Still, individual WLC had lower predictive R^2^ accuracies than other methods (Table 1) because 10 test dates did not sufficiently capture the lactation curve dynamics. Utilizing more test-day records led to better accuracies with WLC. For example, with 20 test-day records, the R^2^ accuracy increased to 0.471 (*ρ* = 0.5), 0.683 (*ρ* = 0.7), and 0.895 (*ρ* = 0.9), respectively, when mastitis was absent, and 0.602 (*ρ* = 0.5), 0.78 (*ρ* = 0.7), and 0.9333 (*ρ* = 0.9), respectively, when mastitis was present.

**Table 1:** Mean and SD of predictive R^2^ accuracies of 305-d milk yields obtained by various statistical methods assuming varied repeatability (*ρ*)

Model^1^	Absence of mastitis			Presence of mastitis		
	0.5	0.7	0.9	0.5	0.7	0.9
	Mean (SD)	Mean (SD)	Mean (SD)	Mean (SD)	Mean (SD)	Mean (SD)
Wood	0.401 (0.038)	0.626 (0.038)	0.879 (0.012)	0.550 (0.022)	0.748 (0.022)	0.921 (0.007)
BP1	0.488 (0.014)	0.676 (0.010)	0.885 (0.008)	0.611 (0.014)	0.771 (0.011)	0.923 (0.004)
BP2	0.487 (0.015)	0.675 (0.008)	0.885 (0.004)	0.601 (0.020)	0.770 (0.012)	0.923 (0.006)
LR1	0.488 (0.015)	0.676 (0.009)	0.885 (0.004)	0.612 (0.026)	0.776 (0.020)	0.925 (0.011)
LR2	0.487 (0.015)	0.675 (0.008)	0.885 (0.004)	0.609 (0.027)	0.774 (0.021)	0.925 (0.013)
RSEM	0.481 (0.018)	0.672 (0.008)	0.883 (0.008)	0.591 (0.030)	0.766 (0.024)	0.920 (0.017)
MLP	0.485 (0.016)	0.674 (0.012)	0.885 (0.010)	0.615 (0.017)	0.777 (0.014)	0.926 (0.009)
RNN	0.486 (0.018)	0.675 (0.010)	0.885 (0.006)	0.619 (0.025)	0.779 (0.024)	0.926 (0.015)

1 Wood = Wood lactation curve; BP1 = best prediction estimating 305-d milk yield through aggregating all the observed and estimated daily milk yield; BP2 = best prediction estimating 305-d milk yield directly from test-day yields; LR1 = linear regression estimating 305-d milk yield through aggregating all the observed and estimated daily milk yield; LR2 = linear regression estimating 305-d milk yields directly from test-day yields; RSEM = recursive structural equation model; MLP = feed-forward multiple-layer perceptron neural network; RNN = recurrent neural network with long short-term memory units.

Best prediction is straightforward to implement, and outperformed other models when mastitis was absent (Table 1), demonstrating that simplicity prevailed when anything else was not involved. When mastitis was present, BP ignoring SCS performed worse than other methods (Table 1). In practice, BP is conducted within environmental or management groups, but grouping all possible health conditions can be challenging or impossible. Alternatively, modified BP can be used (Gillon et al., 2010). In the presence of mastitis, LR outperformed BP, and 2 nonlinear models (FNN and RNN) outperformed the linear models (BP, LR, and RSEM) (Table 1). Arguably, neural networks are universal approximators because every continuous function that maps intervals of real numbers to some output interval of real numbers can be approximated arbitrarily closely by a multiple-layer perceptron. Nevertheless, BP accuracies were more consistent between HCV replicates revealed by their smaller standard deviations. The 2 aggregation approaches, BP1 and LR1, provided better accuracies of estimated lactation yields than their counterparts (Table 1). However, such a conclusion was subject to the simulation of data noises. Overall, the accuracy of estimated daily milk yields was lower for the first 1 to 2 wk and then increased and stabilized approximately till the end of lactation (Figure 1C). Aggregation was not implemented with RSEM, FNN, and RNN due to intensive computing. In theory, the performance of neural networks is broadly tunable, subject to (1) optimal NN structural (e.g., layers of perceptron, neurons, activation functions), (2) training algorithms, (3) regularization, and so on. Therefore, leveraging the optimal structures of NN models is crucial to improve prediction accuracy (see graphical abstract; the bottom graph). The RNN models outperformed FNN models, but the difference became smaller as repeatability increased. Overall, the accuracy differences between these methods were small, except for the RSEM.

Recursive models facilitated inferring causality relationships underlying lactation, but the accuracy varied drastically between the 2 causal models. In the presence of mastitis, RNN had the best accuracy on average (0.59–0.92). For example, Figure 2 shows the R^2^ accuracy, plots of observed against predicted daily milk yields, and trajectories of the loss function (mean square error) and mean absolute error obtained from the first replicate in the third scenario (mastitis absent and repeatability equaling 0.9). The RSEM assuming homogenous recursive effects had the worst average accuracy (0.62–0.93) because it did not precisely capture the causality effects in the present study when mastitis was absent. The estimated recursive effects from a previous to the following DMY were between 0.84 and 1.05. The estimated effects from daily milk yield to SCS on the same milking date were all positive (0.16–0.18). The estimated effects from the previous SCS to the following DMY were also all positive (4.8–6.0). However, by simulation, SCS had a negative effect on the next milk yield when exceeding the subclinical threshold. The reason for this discrepancy was that mastitis instances with SCS ≥5 were low, and the estimated recursive effects from SCS to the following daily milk yield were dominated by healthy animals (SCS <5). For health animals, SCS had positive recursive effects on the following DMY because, by simulation, SCS was linearly correlated with DMY. Another reason could be varied approaches to infer the causal relationships. The structure coefficient matrix in the RSEM assumed an oversimplified joint probability for involving milking variables in the sense that the variable at a later test day depended on the immediately preceding one only:

[9] $$Pr\left(y_{1},y_{2},\ldots ,y_{t}\right)=Pr\left(y_{1}\right)Pr\left(y_{2}|y_{1}\right)Pr\left(y_{3}|y_{2}\right)\ldots Pr\left(y_{t}|y_{t-1}\right).$$

Unlike RSEM, RNN was implemented that captured a joint probability distribution over all the prior states,

[10] $$\begin{array}{l} Pr\left(h_{1},h_{2},\ldots ,h_{t}\right)=\\ Pr\left(h_{1}\right)Pr\left(h_{2}|h_{1}\right)Pr\left(h_{3}|h_{1},h_{2}\right)\ldots Pr\left(h_{t}|h_{1},\ldots ,h_{T-1}\right). \end{array}$$

Many, if not all, statistical models fall into 2 categories, explanatory and prediction. Explanatory models aim to infer causal hypotheses, whereas prediction models focus on predicting new or future observations, irrespective of whether included predictors are causal or not (Shmueli, 2010). As a result, data mining algorithms, such as neural networks, are widely used to capture complicated associations, even though they may not provide insight into the underlying causality. Let Y=F(X) be the true relationship between *Y* and *X*. Let measurable *x* and *y* be the operationalization of *X* and *Y*, respectively. Also, let a statistical model *f* be the operationalization of F, such that *E*(*y*) = *f*(*x*). Because F is usually not sufficiently detailed to lead to a single *f*, a set of models is often considered. Thus, explanatory modeling seeks to match *f* and F as closely as possible for the statistical inference to apply to the theoretical hypothesis. For this purpose, the data *x* and *y* are used as tools for estimating *f*, which is then employed to test the causal hypothesis. In contrast, the predictive model focuses on *x* and *y*, where the function *f* is used as a tool for generating predictions of new *y* values.

**Figure 2:**
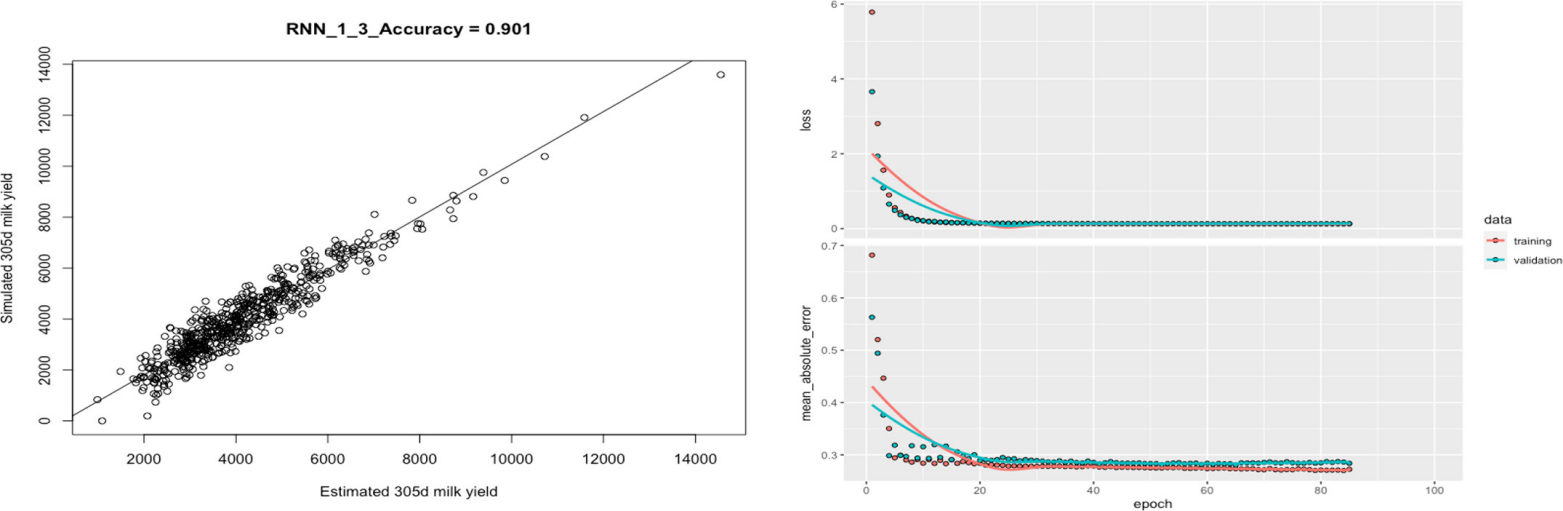
Plots of observed against estimated 305-d milk yields (left) and trajectories of the loss function (mean squared error) and mean absolute error evaluated in the training and validation sets, respectively (right) for estimation of 305-d milk yield using a modified recurrent neural network (RNN) with long short-term memory units (LSTM). The results were obtained from the first replicate and the third scenario (absence of mastitis; repeatability = 0.9).

Because correlational and casual models differ in their goals and modeling strategies, conflation between causal inference and prediction often represents a methodological error. Still, a causal model can practically appeal to prediction if it improves accuracy. So, did causal models improve the accuracy of estimating milk yield in the present study? The answer was yes for RNN, but no for RSEM. Why is that? The answer lies in expected prediction error (**EPE**), which can be decomposed into 3 components using a quadratic loss function (Hastie et al., 2009):

[11] $$EPE=E{\left(y-\hat{f}\left(x\right)\right)}^{2}=Var\left(y\right)+Bias^{2}+Var\left(\hat{f}\left(x\right)\right).$$

The first term of EPE is the error that will occur even if the statistical model is correctly specified and accurately estimated. The second term is the squared bias resulting from misspecifying the model. The third term is estimation variance when using a sample to estimate *f*. Hence, prediction accuracy depends on these 3 components. For the explanatory model, the focus is often on minimizing the bias to obtain the most accurate representation of the underlying theory. In contrast, predictive modeling aims to minimize the combination of bias and estimation variance, occasionally scarifying the theoretical accuracy for improved empirical precision. Depending on the relative quantities of the 3 components, there are situations where an “incorrect” model can predict better than the “correct” model (Shmueli, 2010). It is also possible that a causal model with significantly misspecified causal relationships can predict poorly.

Often, one makes decisions by considering all the factors that might influence the choice, weighed by their relative importance. By doing so, one could overemphasize peripheral variables at the expense of critical ones. On the other hand, simple models could mute unhelpful peripheral variables while amplifying critical ones, possibly leading to a loss of accuracy. While statistical methods of varying complexity are available, a parsimonious model is preferred, subject to the tradeoff between the model complexity and accuracy. In addition to choosing statistical models, it is equally crucial to account for factors and covariates affecting milk yields. Finally, we predicted lactation milk yields based on single test-day yields. However, using *n*-day averages can increase the accuracy by a factor $\frac{n}{1+\rho \left(n-1\right)},$ where *ρ* is between-test-day repeatability (Falconer and Mackay, 1996).
